# “Imposed Motherhood, Interrupted Childhood”: child marriage, postpartum trauma, and the failure of SDG commitments in Chennai's inner city

**DOI:** 10.3389/fgwh.2026.1710482

**Published:** 2026-05-20

**Authors:** J. Priyadharshini, R. K. Jaishree Karthiga

**Affiliations:** School of Social Sciences and Languages, Vellore Institute of Technology, Chennai, India

**Keywords:** adolescent motherhood, child marriage, feminist frameworks, mental health literacy, phenomenological trauma analysis, policy reform, postpartum struggles, SDG 5.35

## Abstract

Child marriage continues to be a deeply rooted tradition in some urban Indian communities, such as Sowcarpet, where cultural conventions, economic vulnerability, and patriarchal norms combine to normalize the early marriage of girls between 12 and 18 years of age. This qualitative study employs a descriptive phenomenological approach to analyze the emotional and psychological consequences of child marriage among eight adolescent brides, using snowball sampling and detailed oral accounts to examine child marriage as a form of sexual and psychological abuse. The analysis identifies five key themes, namely, cultural grooming, emotional isolation, postpartum trauma, power imbalance, and silent resistance. The findings reveal that adolescents experience severe psychological distress, including postpartum depression and symptoms of psychosis, highlighting the terror and confusion faced by girls who are emotionally immature yet compelled into motherhood. These experiences disrupt identity formation and contribute to intergenerational trauma, affecting both the girls' development and their children's wellbeing. This article situates these findings within feminist and trauma-informed frameworks, asserting that child marriage constitutes both a developmental injustice and a violation of bodily autonomy. It further calls for reforms aligned with the Sustainable Development Goals 5.3, 4, 10, and 16.

## 1 Introduction

Child marriage is considered the marriage or union of a child under the age of 18 (1) and remains embedded within various social, economic, cultural, and patriarchal systems that are deeply entrenched in countries all over the world. While international law mandates the elimination of harmful practices against children, child marriage persists and is often normalized through ritual, poverty, and gendered assumptions that devalue girls’ autonomy and wellbeing (2).

Recent studies have focused more and more on the psychosocial price linked to child marriage, particularly focusing on adolescent mothers. Pregnant and postpartum adolescents face heightened health and psychosocial risks, including increased vulnerability to mental health conditions such as depression, compared to older mothers (3). Inadequate maternal emotional wellbeing is linked to compromised child growth, cognitive development, and increased vulnerability to neglect (4). From a health psychology perspective, child marriage is increasingly understood as a critical determinant of mental health, with far-reaching implications for emotional regulation, stress response, and overall psychological wellbeing. As Pujhana (5) highlights, early marriage places young girls in environments characterized by chronic stress, role overload, and limited autonomy, all of which heighten vulnerability to anxiety, depression, and trauma-related disorders. This framing underscores that the impacts of child marriage extend beyond social and cultural dimensions to include profound disruptions in cognitive and emotional development. This study contributes to the existing literature by exploring the lived experience of adolescent brides in a metropolitan South Indian context.

A phenomenological approach is particularly appropriate for this study as it seeks to understand how individuals interpret and make meaning of their lived experiences (6). Descriptive phenomenology focuses on capturing participants’ subjective realities without imposing external theoretical assumptions, allowing the voices of adolescent brides to reveal how early marriage, sexual initiation, and early motherhood are experienced at the psychological and emotional levels. This approach enables a deeper understanding of how cultural practices translate into embodied trauma and identity disruption by foregrounding lived experience. The stories show that child marriage may be perceived as a cultural practice, but it is sexual coercion and psychological abuse, usually ending in postpartum depression, psychosis, and disruption of maternal identity.

The main research question that defines this work is “How do adolescent brides understand child marriage as psychological and sexual trauma, and what are the developmental and intergenerational effects of these experiences?” This study tries to answer the question by first reframing the child marriage issue as a public health crisis and a space of gendered violence, rather than just a cultural phenomenon, through a feminist and trauma-informed lens.

This study aims to raise awareness, promote trauma-informed approaches, and support efforts toward ending child marriage in line with Sustainable Development Goal (SDG) 5.3, while also contributing to broader goals related to health, education, equality, and justice through advocacy for policy reform, mental health literacy, and survivor-centered care. This study contributes to gender scholarship by centering survivor voices and emotional complexity, challenging the cultural normalization of child marriage, and offering insights for recovery, resistance, and policy change.

## 2 Literature review and theoretical framework

Phenomenology as a research approach focuses on the lived experiences of individuals. It can provide deep insights into how they perceive, experience, and resist painful realities (6, 7). In this context, when used to explore child marriage, phenomenology can lead to a better understanding of how adolescents who marry young internalize, navigate, and challenge their roles within exploitative social systems. This section connects important literature in feminist theory, developmental psychology, trauma studies, and global health to frame child marriage as gendered violence with lasting effects across generations.

### 2.1 Phenomenology and the lived experience of child marriage

Phenomenology examines how individuals understand and give meaning to their lived experiences. In the case of child marriage, this approach helps us understand how young brides manage marriage, motherhood, and their identities under the pressures of caste, community, and patriarchy (6, 8, 9). Their stories are shaped by social norms, economic hardships, and gender expectations that support early marriage. The phenomenological view highlights how girls perceive their suffering, often without language, support, or recognition from institutions.

### 2.2 Cultural conditioning and internalized roles

From a young age, girls are conditioned to view marriage as their ultimate fate without considering other possibilities. Child marriage limits girls’ agency by enforcing roles such as wife and caregiver, which often clash with their personal goals and desires (10). These imposed roles shape their identities and strip away concepts such as freedom, self-worth, and empowerment. Internalized gender norms influence women's sense of agency by determining their social value through marriage and caregiving roles within patriarchal households (11). Modern feminist research argues that gender-based violence exists within patriarchal systems that control women's bodies and limit their ability to give genuine consent due to structural inequalities (12, 13).

### 2.3 Sexual abuse and psychological captivity through child marriage

Child marriage is recognized as a form of sexual abuse, especially when girls are married to older men who abuse them and engage in non-consensual sexual acts. These marriages often become coercive due to a lack of emotional readiness and informed consent. Many girls face limited agency and a greater risk of intimate partner violence and negative psychosocial outcomes (14). Feminist theorists view this as psychological enslavement, where girls struggle to grow emotionally and personally (12). Normalizing this abuse within families and communities silences survivors and perpetuates cycles of violence.

Research highlights the psychological effects of child marriage through gendered socialization and the shaping of sexuality. According to Sarfo et al. (15), early marriage enforces strict gender roles that restrict agency and promote submissiveness, influencing girls’ self-image and emotional experiences. These dynamics lead to internalized oppression, lower self-worth, and limited psychosocial development, as young brides confront identities shaped by societal expectations rather than personal growth.

### 2.4 Postpartum depression, psychosis, and maternal identity disruption

Transitioning from child bride to teenage mother brings various forms of psychological distress. Studies show that adolescent mothers are at a much higher risk for postpartum depression and anxiety, especially when pregnancies are unplanned or occur in situations with little autonomy and support (3, 14). These mental health issues can include emotional withdrawal, distress, and challenges in adjusting to motherhood, particularly under coercive and isolating conditions (3). These challenges worsen with early marriage, limited agency, and a lack of trauma-informed care (14).

Developmental psychology emphasizes adolescence as a key period for forming identity, managing emotions, and developing cognitive skills. An early shift into adult roles can disrupt this process, leading to lasting psychological and social consequences (16). Disruptions in forming maternal identity affect not just the mother but also her connection, responsiveness, and emotional attunement to the child, heightening the risk of developmental delays and poor psychosocial outcomes for children (17).

### 2.5 Intergenerational trauma and developmental harm

The psychological challenges faced by adolescent brides impact not only their lives but also the wellbeing and development of their children. Intergenerational trauma refers to the passing down of emotional pain, behavioral patterns, and relationship issues across generations, often linked to unresolved trauma and systemic inequalities. In the context of child marriage, maternal depression, emotional withdrawal, and financial insecurity can create poor caregiving conditions, increasing the risks of neglect, inadequate nutrition, and limited educational opportunities for children (18, 19).

Research in global health and development indicates that children born to adolescent mothers are more likely to experience low birth weight, preterm birth, and poorer cognitive and developmental outcomes (3). Limited access to reproductive healthcare, mental health support, and education exacerbates these risks and perpetuates cycles of disadvantage. In such settings, unresolved trauma and lack of support can lead to emotional suppression and reluctance to seek help, contributing to ongoing vulnerability (18).

### 2.6 Social dynamics and power asymmetries

The social interactions of child brides are influenced by unequal power dynamics and experiences of isolation and loneliness. Research indicates that girls are often married to older men with little decision-making power, and husbands and in-laws enforce gender norms that limit freedom and expression (10, 19). These conditions further marginalize girls within their households and communities.

While peer relationships can provide empowerment, they often shrink due to social restrictions on married girls, limiting their access to support networks and chances for self-expression (10). This loss of peer interaction reduces opportunities for emotional support and collective resistance.

Feminist perspectives illustrate that these situations reflect structural restrictions on agency, where gendered power dynamics limit women's ability to make choices and confront oppressive norms. A lack of access to supportive networks and community accountability can reinforce dependence and weaken self-confidence, making it harder for girls to envision different life paths or seek assistance.

### 2.7 Coping mechanisms and silent resistance

Despite ongoing structural barriers, some child brides show resilience by developing coping strategies that help maintain their self-worth and dignity. These strategies may include creating informal support networks, finding small opportunities for autonomy within restrictive settings, and redefining their roles to find personal meaning in marriage. Such responses can be seen as subtle forms of resistance, where agency is exercised despite limitations. However, not everyone can sustain this resistance; some may internalize dominant social norms and gradually accept their circumstances, resulting in lower expectations for change. In these cases, accommodating emotionally may serve as a survival tactic, as normalizing hardship can lessen personal agency (20, 21).

### 2.8 Educational and economic constraints

The experiences of child brides often reveal significant opportunity costs, especially regarding interrupted education and limited economic engagement. Early marriage is usually linked to dropping out of school early, which hinders skill development and reduces future earning potential, thereby reinforcing long-term economic dependency within marriages. This dependency often maintains existing power imbalances and limits women's ability to make independent choices (22). Research indicates that early marriage and lower educational attainment are associated with poor mental health outcomes, including increased vulnerability to psychological distress and lowered wellbeing (23). Moreover, economic challenges in early marriages can cause maternal stress and negatively affect caregiving conditions, with implications for child development (24). These trends highlight ongoing intergenerational disadvantages, where limited access to education and job opportunities fosters cycles of poverty and inequality. Tackling child marriage requires structural changes to improve girls’ access to education and strengthen paths to economic independence.

### 2.9 Intersectionality and structural violence

An intersectional approach shows that child marriage mainly affects girls who are already marginalized by overlapping social hierarchies, where gender intersects with class, location, and other inequalities, limiting access to education, healthcare, and legal protections (25). These combined disadvantages are not just additive but also mutually reinforcing, shaping girls’ realities in ways that increase vulnerability and limit their ability to assert agency. Research reveals that structural, programmatic, and sociocultural inequalities work together to limit access to vital reproductive and maternal health services, showing how institutional obstacles and social norms sustain exclusion (26).

An intersectional perspective also emphasizes that coercion in child and forced marriage manifests through both subtle and overt controls present in family, cultural, and legal settings, often silencing resistance and making compliance seem normal (27). These influences are experienced differently depending on social position, where stigma, surveillance, and moral scrutiny discipline girls’ actions and restrict their ability to seek support or challenge abuse. Therefore, child marriage should be viewed not just as a social practice, but as a type of gendered violence shaped by interlocked systems of power that lead to lasting psychological, developmental, and intergenerational effects.

Recent studies stress the need for context-sensitive approaches that center the voices of survivors and recognize that structural change requires more than isolated policy actions (26, 27). By highlighting lived experiences in specific social contexts, these perspectives show how early marriage disrupts critical developmental paths and strengthens cycles of disadvantage. This underscores the requirement for research and policy frameworks that incorporate intersectionality, uplift marginalized voices, and address the complex interactions of coercion, structural inequality, and limited access to services in the lives of child brides (25, 26). The methodology section below offers the ethical, contextual, and analytical frameworks used to gather these stories in Sowcarpet, Chennai.

The psychological aspects of child marriage can also be understood through eco-cultural frameworks that place individual experiences within wider cultural and environmental systems. As suggested by Banlanjo (28), cultural norms, family structures, and economic conditions work together to shape psychological growth and limit agency. This viewpoint allows for a better understanding of how environmental factors influence emotional responses, coping methods, and identity development in young girls.

## 3 Methodology

### 3.1 Research design

This research study applied a descriptive phenomenological lens to the real-life experiences of adolescent brides, guided by Colaizzi's method (29). Phenomenology allows for participants’ subjective realities to be deeply engaged with, showing which grounds they interpret, bear, and continue through child marriage in their complex sociocultural and patriarchal environments. This design allows for a trauma-informed understanding of child marriage as both sexual coercion and psychological abuse.

Phenomenology allows us not to view child marriage as just a single event but to also see the ways in which girls deal with the emotional, relational, and developmental impacts over time. The study uses a feminist approach that values lived experiences as the main source of knowledge and seeks to challenge dominant narratives that often hide or minimize the harm caused.

### 3.2 Sampling and participants

The participants in this study were from Sowcarpet, a densely populated commercial area located in North Chennai, Tamil Nadu, known for its wholesale markets and closely connected residential communities. Economic pressures, strong family networks, and traditional community expectations often influence decisions surrounding early marriage in this locality. The snowball sampling technique was employed to select the participants, as it is the most suitable method for accessing the most vulnerable and the hard-to-reach groups. The process was initiated through community contacts who identified potential participants meeting the study criteria. Community health workers and informal networks were used as the initial points of contact, and the participants recruited afterwards were named through referrals. Referrals were sought from multiple starting points rather than relying on a single social network to minimize selection bias. This approach allowed the study to capture a broader range of experiences within the community. Eight adolescent girls aged 12–18 years were chosen based on the following criteria:

1. married prior to their 18th birthday;
2. willing to recount their experiences of marriage, abuse, and motherhood; and
3. capable of participating in interviews conducted in Tamil.

The majority of the participants belong to lower-middle and working-class households, where families were engaged in small-scale, informal employment or service-sector work. This limited, purposive sample was a deliberate methodology decision to focus on depth. Sample size in phenomenological research is based on data richness, not statistical generalizability (6). The aim was to elicit layered stories that highlight the psychological and developmental effects of child marriage, specifically on identity disruption, maternal trauma, and intergenerational harm.

### 3.3 Participant profile

All the participants were girls aged 12–18 years and in grades VII to XII at the time of marriage. They belonged to heterogeneous family types, with the majority from nuclear families and living in economic precariousness. Moreover, the majority had dropped out of formal schooling after marriage and had limited access to healthcare and legal services. A few had given birth prematurely and were primary carers for infants or toddlers.

This case study captures the intersection of educational disruption, family expectations, and socio-economic vulnerability that constitutes child marriage in urban inner-city settings. Although the sample is not generalizable to all child brides, it provides vital insight into how structural violence takes hold in individual lives.

### 3.4 Data collection and analysis

Semistructured, in-depth interviews were conducted in Tamil, using a trauma-informed interview protocol developed to minimize emotional distress and ensure participant safety. The protocol emphasized empathetic listening, non-judgmental questioning, and participant control over the pace and depth of the discussion. Participants were informed that they could pause, skip questions, or terminate the interview at any time if they felt uncomfortable. Questions were phrased sensitively to avoid retraumatization while still allowing participants to narrate their experiences in their own words.

All interviews were conducted by the primary researcher, who has prior academic training in qualitative research methods and experience working with gender and trauma-related topics. Prior to the fieldwork, the researcher undertook preparatory training in trauma-informed interviewing techniques, including strategies for minimizing emotional distress and allowing participants to pause or discontinue interviews if necessary. Participants were approached through community referrals, and informed consent was obtained before each interview. In cases where participants were residing within extended family households, permission to conduct the interview in a private setting was negotiated respectfully while ensuring that participation remained voluntary and confidential.

The key variables under study in this research are the psychological and developmental experiences associated with child marriage, specifically focusing on emotional isolation, identity disruption, postpartum trauma, power imbalance, and forms of silent resistance. These variables are significant as they capture the multidimensional psychological impact of early marriage, particularly during adolescence, a critical period for identity formation and emotional development. The emphasis on these constructs enables a deeper understanding of how child marriage operates not only as a social practice but also as a form of psychological and developmental disruption with intergenerational implications.

To ensure the reliability and credibility of the data, multiple qualitative rigor strategies were employed. Interviews were conducted using a consistent semistructured, trauma-informed protocol, and all data were audio-recorded, transcribed verbatim, and carefully translated to preserve meaning and emotional nuance. Credibility was strengthened through prolonged engagement with the data, repeated review of significant statements, and member checking with participants to validate interpretations. Reflexivity was maintained throughout to minimize researcher bias. Validity and trustworthiness were further enhanced through thematic saturation, triangulation of participant narratives across different referral points, and the systematic application of Colaizzi's method, ensuring that findings remained grounded in participants’ lived experiences.

Interviews took place in private, comfortable places selected by the participants and each lasted from 60 to 90 min. Participants were asked to talk freely about the events that led them to get married, their emotional reactions, and their experiences of motherhood, abuse, and isolation.

Some of the questions from the interview schedule were as follows:

1. “What was your first reaction when you were told you were going to get married?”
2. “What was your feeling the first few days of marriage?”
3. “How has motherhood influenced your thoughts and feelings?”
4. “What do you want others to know about your experience?”

All the interviews were audio-recorded with permission and transcribed verbatim. For analysis, the transcripts were first translated into English. Special attention was given to the emotional tone and cultural nuances throughout the translation process.

Colaizzi's seven-step process for data analysis was employed, with modifications to focus on the survivor's voice and emotional depth (29). The steps were as follows:

1. First, read all the transcripts to understand the content in total.
2. Next, pick out the significant statements that are highly related to trauma, identity, and motherhood.
3. Create meanings from these statements.
4. Arrange the meanings according to the identified themes.
5. Describe in detail each theme based on the available data.
6. Confirm the themes through member checking involving select participants.
7. Present the results as a coherent and integrated discourse.

The phenomenological framework guided both the interview design and the analytical process. The interviews were structured to encourage participants to narrate their lived experiences in their own words, allowing meanings to emerge from personal reflection rather than from predetermined categories. This approach aligns with phenomenological inquiry, which seeks to understand how individuals interpret and make sense of their experiences within specific social and cultural contexts (7). During the analysis, emphasis was placed on identifying significant statements that captured the participants’ subjective interpretations of child marriage, motherhood, and emotional trauma, thereby preserving the experiential essence of their narratives.

To enhance the credibility and trustworthiness of the findings, several qualitative rigor strategies were employed. From the audio-recorded interviews, exact words were kept verbatim to maintain the integrity of lived experience and to emphasize the psychological richness of each account. During the analysis, significant statements were repeatedly reviewed to ensure that interpretations remained grounded in the data. Reflexive notes were maintained throughout the research process to acknowledge the researcher's positionality and minimize interpretive bias. Thematic saturation was reached by the sixth interview, with subsequent narratives reinforcing existing themes rather than generating new ones. The main themes that emerged from the data were identity disintegration, postpartum trauma, emotional repression, power imbalance, and silent resistance.

Member checking was conducted with select participants to validate the thematic interpretations. These participants were invited to review summarized interpretations of their narratives and confirm whether the themes accurately reflected their experiences. Minor clarifications provided by the participants were incorporated into the final thematic structure. Although alternative qualitative approaches such as thematic analysis were considered during the research design stage, Colaizzi's phenomenological method was selected because it offers a systematic process for extracting experiential meanings while preserving the authenticity of participants’ voices.

## 4 Ethical considerations

Among other issues, ethical sensitivity was the leading concern throughout the whole study. Approval from the ethics committee was obtained through the School of Social Sciences and Languages, VIT Chennai. Since the emotional vulnerability of adolescent brides was an acknowledged fact, the research design used trauma-informed ethics, which gave priority to safety, consent, and empowerment (30). Given the sensitive nature of the interviews, several measures were taken to ensure the participants’ emotional safety. The participants were informed that they had the right to withdraw at any time and the interviews were conducted in private spaces to ensure confidentiality and the emotional security of the participants. If participants exhibited signs of distress, the interview was paused and emotional support was provided through empathetic listening and reassurance.

None of the participants’ identities was revealed as each was assigned a coded identifier (P1–P8), and the corresponding data were stored in a secure location. In addition to providing them with emotional support, they were given directions to counseling institutions that offer the service in case they needed help.

The research was reviewed and approved by an independent ethics committee, and all procedures adhered to national regulations for research on minors and survivors of gender-based violence. Parental consent was not obtained because married adolescents have an emancipated status, although verbal assent was sought from every participant, and caution was exercised to provide full voluntary and informed participation.

This approach demonstrates an adherence to feminist research ethics, grounding marginalized voices, honoring emotional labor, and creating knowledge that can be used to inform advocacy and policy change.

## 5 Findings and thematic analysis

This phenomenological study of eight adolescent brides in Sowcarpet, Chennai, yielded five core interconnected themes that map the psychological, emotional, and developmental impact of child marriage. These themes surfaced via Colaizzi's method, which allows the survivor's perspective, the emotional gist, and the contextual breadth to be presented. These did not emerge as isolated experiences but rather as overlapping dimensions of the participants’ lives. The results indicate that child marriage is one of the many faces of sexual and psychological abuse, the effects of which reach far beyond that of motherhood, identity, and even intergenerational wellbeing (Table 1).

**Table 1:** Themes derived from of the findings.

Group(s)	Theme	Narrative
(a) Exploring the idea of getting married (b) Accepting to get married (c) Entering the marital cord	Inquisitiveness	Their peers getting married at their age makes them inquisitive to do the same thing without thought. Marriage is romanticized, and curiosity replaces informed consent.
No sharing of experience with already married peers	Solitude	During plans of getting married, they are not able to talk to their friends directly. Hence, their chance of knowing the experience of their friends is completely removed. Emotional isolation begins before marriage and deepens after.
(a) Anxiety of sharing the issue (b) Anxiety of guilt (c) Thinking of family status (d) Could not understand it as major child abuse	Vulnerability	Feeling very weak, they do not rebel or seek professional help. Fear, guilt, and emotional suppression prevent disclosure. They internalize abuse as duty.
(a) Physical and mental abuse (b) Stress and trauma (c) Staying disconnected (d) Sexual coercion (e) Postpartum depression and psychosis (f) Fear of mothering while still a child	Suffering	After getting married as a child, the burden of responsibilities makes them feel anxious and naturally helpless. They experience sexual trauma, identity disintegration, and maternal distress.
(a) Sex education (b) Be careful when making life choices (c) Educate on child marriage and its severe consequences (d) Legal reform and community action	Precautionary strategies	To benefit others, they need to sensitize school-going girls and make a clarion call. Survivors advocate for education, awareness, and systemic change to prevent future harm.

The participants described their experiences in their own words and the interviewer employed open-ended guide questions in order to keep participants on track and the flow intact. The participants’ narratives suggest a progression in which early cultural conditioning normalized child marriage, emotional isolation intensified vulnerability during pregnancy and early motherhood, postpartum trauma revealed the psychological consequences of these conditions, and entrenched power imbalances within marital households limited women's agency. Despite these constraints, the participants also described subtle forms of silent resistance, demonstrating their attempts to navigate and reinterpret their circumstances.

Sample guide questions used in the interviews are given below under each theme.

### 5.1 Inquisitiveness: grooming and cultural conditioning

The interviewer asked the following:

1. What did you think about marriage before it happened?
2. How did you come to understand what marriage is?
3. Did anyone talk to you about what to expect?
4. What made you feel curious or excited about marriage?

The first theme that emerged from the stories is how girls are gently shaped to view child marriage as a normal, desirable, and even aspirational life experience. Participants described how marriage was idealized in their communities, seen as a rite of passage, a sign of maturity, and a way to gain social acceptance. This early cultural conditioning came mostly from observing peers, family rituals, and media that portrayed marriage as a joyful milestone rather than a significant commitment.

Early socialization and peer observation also shaped how participants perceived marriage before experiencing its realities. Participant 2 reflected, “I saw my friends getting married and wondered what it would be like. Everyone talked about how it's part of growing up, and I wanted to feel grown-up too” (P2) illustrating how marriage was framed within the community as a marker of maturity and social belonging. Similarly, another participant recalled, “I thought marriage was about happiness, and I was curious to find out what it meant. No one told me what would actually happen after the wedding” (P5), revealing how idealized narratives of marriage concealed its practical and emotional consequences. A comparable sentiment emerged in another account, where a participant noted, “I saw how much attention brides get, and I wanted to feel that. Everyone said it was a happy moment, so I thought it would be fun” (P7). Together, these reflections demonstrate how cultural celebration and peer influence normalized early marriage while obscuring its long-term implications.

These comments imply a significant deficiency in the participants’ knowledge about the topic. The desire to know, comparing oneself to peers, and the lure of a party with friends replaced the understanding of the matrimonial union with something greater than its sexual, emotional, and legal components. Moreover, the respondents stated that not only could they not ask questions, but they also could not listen to the experiences of others due to social stigma or emotional suffering. This silence created a vacuum in which cultural myths thrived.

Feminist scholars propose that such grooming is a demonstration of patriarchal dominance over women's bodies and futures, in which silence and obedience are privileged and agency is systematically undermined (12, 43). Girls are not simply passive recipients of tradition; they are positively conditioned to internalize the wife and mother roles as their destiny. This conditioning typically starts early, through gendered expectations, ritual activities, and moral scrutiny that undermine personal autonomy and critical thinking.

The tendency among adolescents to romanticize marriage is just one aspect of this phenomenon. Studies conducted in South Asia and sub-Saharan Africa have found the same pattern of adolescent girls seeing marriage as a source of comfort, a stepping stone to prestige, and a way out of poverty or parental control (31). But frequently, many girls are hit by the reality of a patriarchal atmosphere, sexual abuse, the repression of feelings, and continuous caring for the house without any prior preparation or support.

Marriage during adolescence interrupts the process of identity formation, emotion regulation, and cognitive development, forcing girls to enter adult life with their own psychological growth still unresolved. This leads to identity confusion, emotional turmoil, and a higher risk of mental health illness. The incidents recounted here shed light on how cultural socialization conceals the issue of consent and slows down progress. As participant 4 reflected, “No one told me what marriage really meant. I thought it was like a festival, but it became something I couldn't understand” (P4) and participant 6 stated, “I didn't know what sex was. I didn't know what being a wife meant. I just followed what they instructed me” (P6).These statements illustrate how the participants entered marriage with a limited understanding of its implications, revealing how cultural narratives surrounding marriage can conceal issues of consent and preparedness.

These observations highlight the emotional confusion that comes with culturally approved grooming. Girls are not provided with the means to perceive or to resist; they are taught to comply, often in the interest of family honor or protection. Child marriage transgresses that right by substituting duty over self-determination.

The theme also resonates with SDG 5.3, which demands the eradication of harmful practices such as child, early, and forced marriage. One of the obstacles that prevents people from realizing such a dream is cultural conditioning, which, inter alia, leads to the suppression of the voices of the opposition to the practice of violence. This calls for legal reform but also community-level interventions that challenge these myths, engage in dialogue, and empower girls to question inherited beliefs.

This inquisitiveness theme shows that child marriage is not merely enforced; it is internalized through coercive cultural grooming in the guise of celebration. The emotional and developmental impact of this conditioning is deep, laying the groundwork for the isolation, trauma, and resistance that are mapped in later themes.

The narratives suggest that early cultural conditioning did not merely normalize child marriage but also prepared participants to accept unequal marital roles without question. This early socialization often laid the foundation for the emotional isolation experienced after marriage, as participants internalized expectations of silence, obedience, and endurance.

### 5.2 Solitude: emotional isolation and silencing

The questions asked by the interviewer were as follows:

1. How did your relationships change after marriage?
2. To whom do you speak when you are sad or puzzled?
3. Can you describe the experience of trying to communicate your feelings?
4. Do you get listened to and understood in your current place?

The second theme discovered through the stories is the intense emotional alienation experienced by adolescent brides both prior to and after marriage. Though child marriage was presented as a protective or stabilizing measure, the participants reported a profound feeling of loneliness, invisibility, and emotional abandonment.

This isolation began during the planning stages of marriage, when girls were discouraged from discussing their fears or asking questions. It worsened after marriage, when they were removed from peer groups and placed in hierarchical family structures. One participant expressed a deep sense of separation from her social world, stating, “I miss my friends… It's been ages since I saw them. I can hear them having fun outside, but I am not allowed to go” (P1), illustrating how restrictions on mobility severed their connection with peers. Another participant described the loss of personal agency within the marital household: “No one asks me what I want. They just say this is my duty now, but I wasn't the one who chose it” (P6). Similarly, emotional neglect was reflected in another account, where a participant stated, “There are times when I just want to cry, but no one is here to hear. No one would care if I were cheerful or sad” (P8). Together, these narratives reveal how emotional isolation was embedded within everyday marital expectations.

These reflections show that emotional isolation is not a coincidence; it 's a systemic issue within the institution of child marriage. Girls are expected to suppress their feelings, take on the roles of adults, and do the housework without showing any signs of dissatisfaction. Their emotional needs are trivialized as being childish or inconsequential, and their voices are subordinated to those of in-laws, husbands, and elders in society.

Emotionally ventilating in male-dominated systems is often considered a protest or frailty, especially if it is contrary to conjugal standards. Such silencing amplifies psychological distress, identity disintegration, and the internalization of shame. One participant explained this internalized fear of judgment: “I wanted to know from my friend what it was like, but I was scared. What if they thought I was bad for asking?” (P4). Another similarly described remaining silent despite intense fear, stating, “I didn't know how to say I was scared. I just kept quiet and hoped that the fear would go away” (P2). These accounts highlight how moral surveillance and social expectations discouraged girls from articulating their anxieties.

The anxiety and guilt that come with emotional suppression are the focus of these comments. The girls were afraid of judgment, rejection, and moralistic advice, even from their friends. Not being able to share or handle feelings of fear and confusion made the space in which they continued to grow. This conforms to trauma theory, which points to the impact of relational disruption and emotional repression as major contributors to depression, anxiety, and dissociation development (44).

Life from school to marriage is an example of a sudden and drastic change in social connectedness. The participants explained that they lost contact with friends, teachers, and mentors, individuals who could have provided emotional support or alternative views. This loss was compounded by the absence of institutional support, including counseling services, peer groups, or safe spaces for adolescent girls. One participant reflected on this loss, noting, “I used to talk to my teacher when I was sad. After getting married, there was no one like that” (P3)*.* Another expressed a longing to return to familiar spaces of belonging, explaining, “I wanted to go back to school, just to see my friends. But they said I had responsibilities now” (P7). These narratives illustrate how early marriage disrupted the social networks that typically support adolescent development.

Emotional isolation at the public health level is a risk factor that leads to mental illness such as depression, anxiety, and suicidal ideation (32). In the context of child marriage, these risks are magnified by the absence of mental health literacy, stigma surrounding emotional distress, and inaccessibility to care. Participants said they saw themselves as invisible, that they could not communicate, and that they felt emotionally numb, from which they usually experienced severe psychological symptoms. As one participant expressed, “It was like I vanished. Nobody tried to find me” (P6). Another participant similarly described emotional withdrawal, stating, “I shut down. I had no idea what to say and nobody bothered me” (P5). These experiences suggest not only a loss of voice but also a loss of social recognition and care.

Not only are girls denied a voice, but also visibility, empathy, and care. This lack of visibility nurtures dependence and erodes self-esteem; thus, it is harder for girls to ask for support or to envision new paths.

The theme of solitude illustrates how child marriage operates as a form of emotional exile. Girls are removed from their belongingness, restrained in their personal spaces, and emotionally starved for the necessary support to be able to survive and flourish. Their suffering is not only of a psychological nature, but it is also relational, developmental, and systemic. These aspects force the recovery concept to be changed, in which the emotional part of the doctor-patient relationship is central to the sex justice and the healing journey of the victim.

This emotional isolation theme corresponds with SDG 3 (Good Health and Well-being), which demands universal access to mental healthcare and emotional care. It also overlaps with SDG 4 (Quality Education), as school settings often provide safe spaces for emotional growth. Addressing emotional isolation requires trauma-informed approaches that focus on relational healing, peer connections, and amplifying survivor voices.

Emotional isolation rarely occurred in isolation from other challenges. The absence of emotional support within marital households frequently intensified the participants’ psychological distress during pregnancy and early motherhood, thereby deepening the experience of postpartum trauma described by many participants.

### 5.3 Suffering: postpartum trauma and maternal identity disruption

The following interview questions were aimed at assisting respondents in sharing their postpartum experience:

1. How did you feel when your baby was born?
2. What were your thoughts during the first few weeks as a mother?
3. Did you expect to become a mother?
4. How did other people react to your emotions after childbirth?
5. Are there any similarities or differences between your experience and other people's?

The third of the themes that unfolded through the stories is the deep psychological and emotional trauma of adolescent brides both during and after giving birth. The participants spoke of symptoms indicative of postpartum depression, psychosis, and dissociation, where states of being interfered with their capacity for bonding with their babies and eroded their sense of identity. The transition from adolescence to motherhood was sudden, disorienting, and emotionally overwhelming, and it often happened unprepared, unsupported, and without reproductive awareness.

Several participants described intense psychological distress following childbirth. One participant reflected on a loss of self and intrusive auditory experiences, explaining, “After the baby came, I stopped feeling like myself. I couldn't sleep. I kept hearing voices telling me I was a bad mother” (P3)*.* Another participant described fear and emotional unpreparedness for caregiving, stating, “I was afraid to touch the baby. I thought I'd drop her. I didn't feel ready. I wasn't ready” (P6). Similarly, another participant recalled experiencing emotional numbness rather than joy, explaining, “I didn't want to hold my baby. I felt numb. Everyone said I should be happy, but I felt like I was drowning” (P7)*.* These accounts illustrate the emotional fragmentation that can accompany coerced or premature motherhood.

The participants reported feeling disconnected from their bodies, feeling overwhelmed with responsibility, and being afraid of hurting their children. The symptoms of postpartum depression and psychosis observed during the interviews were further worsened by isolation, lack of reproductive education, and the absence of trauma-informed maternal care. For instance, one participant described experiencing auditory misperceptions and fear of her own reactions: “Sometimes I thought the baby was crying even when she wasn't. I was scared of myself” (P5). Another expressed anxiety regarding basic caregiving tasks, explaining, “I didn't know how to feed her. I was scared I'd hurt her. I kept asking, ‘Why did they make me a mother?’” (P1). Similarly, another participant described a persistent sense of developmental incongruence, stating, “I still wanted to play. I didn't know how to be a mother. I felt as if I were faking it” (P8).

These remarks highlight the developmental mismatch between adolescent psychology and maternal responsibility. If young girls are made mothers ahead of time, they will not be able to finish the above-mentioned psychological tasks; hence, their psychological development is curtailed, which is followed by identity fragmentation and emotional deregulation.

Attachment theory exemplifies the disruption in the case of maternal-infant connections. If adolescent mothers are depressed or psychotic, they struggle to connect with their babies. This increases the chances of insecure attachment, developmental delay, and emotional neglect. These effects are not short-lived, as they extend into adolescence and adulthood, generating cycles of trauma and vulnerability.

One participant expressed this sense of detachment by stating, “I didn't feel like I was the baby's mother. I felt like I was babysitting someone else's child” (P4). Another participant described the overwhelming pressure of expectations placed upon her, explaining, “I wanted to run away. I didn't know how to become the person that they expected me to be” (P2). These narratives illustrate how early motherhood can destabilize maternal identity when it occurs in contexts of coercion and emotional distress.

These considerations point to the collapse of the mother's psychological dimension, which is a major element of mental health. Motherhood ceases to be a source of empowerment and becomes a source of trauma, and maternal identity takes form through fear, guilt, and suppression of feelings.

Lack of mental health support escalates the problems mentioned. The participants revealed that their suffering was overlooked, misinterpreted, or spiritualized. Some were instructed to pray; others faced accusations of weakness or ingratitude. One participant explained, “They said I should be grateful. But I felt broken. I didn't know how to explain it” (P6). Another participant described concealing her distress due to fear of stigma, stating, “I thought I was going crazy. But I kept it to myself. I was afraid they would say that I was under a curse” (P3). None received access to counseling, psychiatric services, or peer support. This aligns with global research findings showing that teenage mothers in deprived settings are seldom checked for postpartum disorders and consequently quietly continue in their misery (32, 42).

These instances emphasize the need for a trauma-informed approach to maternal care, especially for adolescent mothers. Screening for postpartum depression and psychosis, receiving counseling that is culturally sensitive, and having peer support groups form the basic elements of recovery. In their absence, girls are trapped in the patterns of emotional distress, unable to care for themselves or their babies.

This issue corresponds with SDG 3 (Good Health and Well-being), which demands universal access to mental health and maternal care. Moreover, this element is directly linked to Sustainable Development Goal 16 (Peace, Justice, and Strong Institutions), as adolescent mothers becoming psychologically damaged due to a lack of protection is proof of systemic neglect and institutional failures. Postpartum trauma can only come to an end through healing, not just through the availability of clinical resources but also through a cultural shift where adolescent motherhood is seen as a position of vulnerability rather than one of moral failure.

One participant's insight about seeing herself in her daughter and her mother reflects a moment of emotional clarity and deep grief. This highlights the need for survivor-focused interventions that break these cycles through education, mental health support, and community accountability.

“I see my daughter and wonder if she will experience what I did, that is, being confused, scared, and not knowing what her life is meant to be. I don't want her to feel lost like me.” (P8)

Such a statement points to the emotional aspect of adolescent motherhood, which is present not only in the existing situation but also in the fear of it being repeated in the next generations. This requires intergenerational healing processes whereby the trauma is recognized, identified, and stopped.

This theme of suffering makes clear the ways in which child marriage results in maternal trauma, identity disruption, and intergenerational harm. These girls are not merely deprived of childhood themselves; they are deprived of the emotional scaffolding to become mothers. Their suffering is not merely personal, but it is structural, developmental, and highly gendered. These findings necessitate a reimagining of maternal care, in which the survivor’s voice and emotional truth are at the center of healing and justice. The participants’ descriptions of postpartum distress were closely intertwined with the structural power dynamics of their marriages. The lack of autonomy, limited decision-making power, and dependence on spouses or in-laws often prevented participants from seeking emotional or medical support, reinforcing the broader patterns of power imbalances discussed in the following theme.

### 5.4 Vulnerability: power imbalance and entrapment

The interviewer's guiding questions were as follows:

1. How old was your husband when you got married?
2. Did you have a choice in the marriage?
3. What happened when you tried to assert yourself?
4. How do you experience control or dependency in your marriage?
5. What does safety mean to you now?

The fourth theme that the participants’ stories brought to light is the influence of the power imbalance that characterizes child marriage as a system that emotionally imprisons the children. The participants described their marriages as those characterized by significant age differences, financial dependence, and the lack of autonomy. Their likes, rights, and emotional requirements were habitually disregarded, while some met with sexual coercion, rape by their spouse, and domestic violence. The weddings were forced most of the time, with the pretense of protection or family honor. Reflecting this, a few participants stated the following:

“He was 30. I was 14. I didn't know how to say no. I didn't even know I could.” (P5)

“He touched me before I even knew what that meant. I didn't know how to say no.” (P4)

“They told me I was fortunate to get married early. However, nobody asked if I was prepared for it. I was frightened at every night.” (P6)

The reflections here hint at the lack of consent and the emotional numbness resulting from force. The participants recollect their very young age when they could not even attempt to understand the notions of sexual closeness and set limits for themselves. Without their knowledge, their bodies were places of control, and their silence was considered agreement to the acts. Feminist scholars maintain that these relationships are typical of sexual violence and psychological slavery, where young girls are deprived of the right to bodily and emotional freedom (12).

This power difference was not limited to the sexual aspect but extended to the structural side. The girls talked about being dependent on their husbands and their families for money and not having any personal income, access to education, or legal rights. This reliance on the other strengthened their insecurity and made them very reluctant to ask for assistance or think about running away. As one participant expressed. “I feel like I'm caged. Everyone makes decisions for me, and I'm clueless about the way out” (P7), highlighting how there is no power to make decisions on their own. Another participant stated, “There is no one to protect me, no one to fight for me. It is as if I am all alone in this” (P2), which underscores the entrapment.

The above remarks disclose the emotional imprisonment that is common in child marriages. The girls are isolated from other children, not allowed to speak inside their homes, and are not provided with the necessary skills to handle their pain. When the girls are abused, they usually cannot or dare not raise their voices out of fear, shame, and distrust of the systems that are supposed to help them.

The narratives also depict how cultural norms intensified the confinement. The girls were informed that being married at a young age was an advantage, a protection, or a good deed. These proclamations became so ingrained that it was difficult for them to separate abuse from responsibility. Incidents of oppression were equated with the human condition, particularly in the case of sexual and emotional abuse, such that the fight for their rights was practically non-existent. The statement by one participant that “I didn't understand what was happening to my body. I just cried and stayed quiet” (P2) reflects how the adolescent could not differentiate the abuse and oppose it. Another participant voiced, “They told me that this is what every girl experiences. So I thought maybe I was just weak” (P6), which highlights the normalization to keep them both physically and mentally vulnerable.

These recollections portray the emotional bewilderment and the tendency to blame oneself that usually go with coercive atmospheres. In addition to a lack of agency, the girls were also deprived of the words needed to express their suffering. To negate one's grief is a manifestation of the patriarchal system that operates through the enforcement of silence and the moralizing of suffering.

One participant's reflection on her mother's experience adds a powerful layer of intergenerational trauma:

“My mother is only 35 and already a grandmother. She looks at me and sees herself. She says she still doesn't know what her life is for and now she worries about my daughter too.” (P6)

This point, along with the rest of the text, reveals the cycles in which trauma caused by child marriage is passed down from one generation to the next. A mother, whose childhood was marked by early marriage, has had no chance to develop both her personality and emotional health properly.

The power imbalance and entrapment theme aligns with SDG 16 (Peace, Justice and Strong Institutions), which demands the protection of vulnerable people and the elimination of gender-based violence. It also intersects with SDG 5.3, which aims at the elimination of child, early, and forced marriage. Addressing this theme requires legal reform, survivor protection, and community accountability, ensuring that girls are not only heard but also safeguarded.

The vulnerability theme shows how child marriage acts as a system of control rather than care. The girls are forced into positions they cannot decline, silenced in households they cannot escape, and blamed for pain they cannot define. Their suffering is not accidental, but it is structural, sexual, and intergenerational. This, therefore, serves as a reminder of the necessity to create a new understanding of protection that is more human and wherein autonomy, consent, and emotional safety are regarded as the basic rights of the person.

Despite these unequal power structures, the participants’ narratives also revealed subtle forms of negotiation and resistance. While many women outwardly complied with familial expectations, their reflections suggested quiet attempts to reclaim agency within restrictive environments.

### 5.5 Precautionary strategies: silent resistance and survivor wisdom

The interviewer's questions were as follows:

1. What would you recommend to other girls?
2. Have you found ways to cope or resist?
3. What do you want people to understand about your experience?
4. What are your hopes for your daughter or younger girls?
5. How do you express yourself when no one is listening?

The last theme identified in the stories is the participants’ quiet resistance and changed perspective. The participants managed to show through their narratives the qualities of endurance, agency, and the longing to help others in spite of various obstacles such as emotional repression, economic dependence, and social isolation. Their memories not only bring up the pain connected to child marriage but also the wisdom that comes with overcoming it. This debunks the myth of girls being nothing more than victims but expounds upon them as theorists of their own experience who can invent and support social changes.

Several participants emphasized the transformative potential of education, reflecting on the opportunities they felt had been denied to them. One participant expressed regret over the interruption of her schooling, stating, “If only I could have stayed in school… Education could have saved me. Girls should continue studying, not be forced into marriage” (P1). Similarly, another participant framed autonomy as a fundamental right, explaining, “Girls ought to have the power to refuse and be accepted. It is our future, not theirs to decide” (P3). In some cases, participants actively attempted to reclaim educational spaces despite the restrictions placed upon them. As one participant explained, “I taught myself to read again. I have books hidden under my mattress” (P8). These accounts illustrate how education emerged as both a symbol of lost opportunity and a pathway toward personal empowerment.

Such statements reveal a deep understanding of the systemic damage alongside a clear prevention vision. The participants pointed out that education, legal literacy, and community responsibility are the most important strategies to bring about change.

The act of teaching oneself to read, hiding books, or imagining alternative futures represents a form of silent resistance, small but powerful gestures that reclaim agency in environments designed to suppress it. These approaches challenge deficit-based narratives that portray child brides as voiceless or broken. They, rather, manifest the emotional acumen, the strategic mindset, and the commitment to safeguard the coming generations of youth.

Participants’ reflections also revealed deep concern about the possibility of intergenerational repetition. One participant described looking at her daughter and fearing that the same confusion and loss of direction might shape the child's future, stating, “I look at my daughter and wonder if she’ll feel the same way I do… confused, scared, not knowing what her life is meant to be. I don't want her to feel lost like me” (P8). This self-examination captures the anticipatory grief and defense instinct that so often mark adolescent mothers. The participant's wish to break the cycle of trauma reflects the intergenerational imperative of child marriage. Their voice becomes a guide for healing, not just for herself, but for her daughter and others like them.

In places where opposition is met with repression or silence, an act of questioning the norm would automatically become a revolt. Through this research, the participants proved that resistance is not necessarily of one kind only; people can show resistance by not letting go of the past, opting to talk, or just living with honor.

One participant described advising a younger relative to delay marriage, explaining, “I told my younger cousin not to get married early. I said, ‘Wait. You have time. Don't rush’” (P2). Another participant described using personal writing as a way of processing her experiences, stating, “I started writing in a notebook. Just my thoughts. I don't show it to anyone, but it helps” (P4).

These gestures, namely mentoring, journaling, and advising, are acts of emotional labor and political consciousness. They represent a wish to transform suffering into shelter and silence into a plan. Such revelations are extremely valuable when creating interventions that are not only efficient but also respect the survivor's agency.

This aspect can be strongly linked to SDG 4 (Quality Education), SDG 10 (Reduced Inequalities), and SDG 5.3 (Elimination of harmful practices). The participants’ demand for education, autonomy, and legal reform depicts a comprehensive picture of child marriage prevention through empowerment, empathy, and structural change rather than just through laws.

Importantly, these voices challenge the notion that survivors must be “rescued” or rehabilitated. Instead, they assert that survivors are already theorizing, resisting, and imagining better futures. The work of institutions, policymakers, and researchers is not to represent them, but to listen, amplify, and take action.

One participant clearly articulated, “I don't want pity. I want people to understand. We’re not weak. We’re just not given choices” (P6). This one sentence summarizes the survivor wisdom ethos. It requires recognition, not rescue. It requires solidarity, not saviorism. And it says that change comes from not listening to distant information but to the emotional realities of those who lived the damage.

This cautionary theme conceals in the silence of child marriage a reservoir of wisdom, resilience, and resistance. These girls are not only survivors but also educators, advocates, and visionaries. Their voices provide not only testimony but transformation. To pay homage to them is to act on their wisdom, and to create a world where no girl is forced to survive or be silent.

These acts of silent resistance highlight the complex ways in which the participants navigated oppressive circumstances. Rather than representing complete submission, their narratives reveal ongoing efforts to cope with and reinterpret their experiences of early marriage, motherhood, and psychological distress.

## 6 Discussion

This research shows that child marriage in Sowcarpet, Chennai, is not just a cultural practice or an economic necessity. It is a form of gendered violence that includes sexual abuse, psychological trauma, and disruptions to development. The experiences of adolescent brides expose the emotional, cognitive, and maternal costs of being forced into adult roles while still children. These stories contradict the dominant views that see child marriage as a protective measure or an unavoidable event. Instead, they emphasize the violations of autonomy, consent, and emotional development that are central to these marriages.

The theme of grooming and cultural conditioning highlights how early marriage is constructed and normalized by deeply rooted cultural beliefs and practices. Girls often grow up in settings where marriage is seen as an expected and desirable path in life. These views arise from community norms that prioritize family honor, economic factors, and the idea of a “good match” (33). In these situations, decisions about marriage are rarely personal; they are shaped by collective expectations. Factors such as dowry practices, poverty, and social pressures come together to promote early unions. This normalization can hide the coercive elements of child marriage, making it appear as a culturally accepted transition.

From an eco-cultural viewpoint, early marriage persists through the interplay of cultural values, environmental conditions, and social structures that reinforce its legitimacy (28). These elements shape girls’ views and goals from a young age, limiting informed choices and reinforcing acceptance of predefined roles. As a result, cultural conditioning works not just at the individual level but also through larger community systems that reproduce and support early marriage. These insights point to the need for culturally aware interventions that engage with community norms and address the social and economic factors that keep the practice alive (28, 33).

Several participants shared feelings of emotional isolation and a constant sense of being silenced. They described how marriage restricted their ability to speak freely and reshaped their social realities. The loss of friendships, withdrawal from school, and limited emotional expression created a deep sense of emptiness and increased their distress. These patterns of internal suffering and challenges in seeking help are similar to findings by N-Yelbi (34), who also highlight how survivors of child marriage face social disconnection and emotional suppression in stigmatized, low-awareness settings.

The theme of postpartum trauma and disruption of maternal identity is very important. Participants described experiences linked to postpartum psychological distress, which included emotional numbness, fear, and challenges in adjusting to motherhood. These difficulties were often made worse by social isolation, poor reproductive health knowledge, and a lack of adequate support systems. Evidence shows that early marriage greatly disrupts the formation of maternal identity, especially when combined with low maternal self-efficacy and insufficient family support, both of which are crucial for developing positive maternal roles (35). A systematic review also points out that child marriage negatively impacts maternal identity development and reproductive health outcomes, highlighting the vulnerability of young mothers as they navigate postpartum challenges (36).

While the psychological burden is substantial, some individuals may find resilience and meaning over time. This is reflected in studies on posttraumatic growth following early and forced marriages (45). Collectively, these findings stress the urgent need for focused maternal care interventions for adolescent mothers. This includes early psychological assessments, counseling that fits their context, and improved social support systems to promote healthier identity development and maternal wellbeing.

The participants’ accounts of sexual coercion, economic dependence, and lack of consent reveal deep power imbalances and a strong sense of entrapment in marriage. Many described relationships with significantly older men, where their personal boundaries were ignored, and bodily autonomy was taken away. The lack of effective legal enforcement and community accountability allowed conditions that fostered marital rape and emotional confinement. Such relationships can be seen as a form of psychological captivity, where young girls are denied opportunities for emotional growth, autonomy, and self-determination.

The narratives in this study also highlight the intergenerational consequences of child marriage and adolescent motherhood, particularly in how early marital experiences shape patterns that extend across generations. These accounts align with findings that child marriage is often sustained by intergenerational influences, where familial, social, and cultural factors reinforce its continuation over time (37). In this way, the impacts are not limited to individual experiences but are embedded within broader cycles that affect subsequent generations and social structures. When adolescent mothers experience depression, trauma, or emotional isolation, their capacity to provide consistent emotional attunement may be compromised, which can affect the child's sense of security and early socioemotional development. In addition, the structural conditions associated with child marriage, such as interrupted education, economic dependency, and limited social mobility, may reduce children's access to educational opportunities and supportive learning environments. Consequently, the participants’ experience reflects not only their individual suffering but also broader cycles of disadvantage that may extend across generations unless targeted social and mental health interventions are implemented. This cyclical suffering calls for survivor-centered justice mechanisms and trauma-informed policy reforms that are in line with SDG 16.

The findings of this study support broader evidence that child marriage affects mental health through gendered pathways. John et al. (38) show that experiences like intimate partner violence, limited autonomy, and unequal power dynamics are closely tied to poor mental health outcomes, such as depression, anxiety, and emotional distress. These pathways reveal how structural gender inequalities are absorbed at the psychological level, creating cycles of vulnerability and restricting recovery and wellbeing among young women.

However, alongside these stories of harm, the theme of silence and survivor wisdom also highlights the power and resilience of adolescent brides. Participants described agency through simple actions, such as self-taught literacy, journaling, and mentoring younger girls. These actions helped them reclaim their dignity and seek financial protection for themselves and others. Their calls for education, legal change, and community accountability show a deep understanding of systemic failures and a desire to break cycles of harm. The data clearly reflect SDG 4 (Quality Education), SDG 10 (Reduced Inequalities), and SDG 5.3 (which aims to end child, early, and forced marriage by 2030). Furthermore, these voices challenge negative narratives and recognize the evolving nature of survivor advocacy shaped by the social justice movement. Trauma-informed mental health interventions are particularly critical in contexts such as South India, where adolescent mothers often have limited access to psychological services (18). Integrating maternal mental health screening into existing primary healthcare systems could provide an important entry point for early identification and support. Community-based health workers, including accredited social health activists and Anganwadi workers, already play a significant role in maternal and child health services through India's integrated Reproductive, Maternal, Newborn, Child and Adolescent Health (RMNCH + A) framework (39). It integrates adolescent health within maternal and child health services and provides an existing institutional platform through which trauma-informed psychological support for adolescent mothers could be implemented. These frontline workers could assist in identifying symptoms of postpartum depression, anxiety, and trauma among adolescent mothers and facilitate referrals to appropriate support services with appropriate training in trauma-informed care and mental health literacy. In addition, peer-support groups and community-based counseling initiatives have been shown to improve maternal wellbeing by reducing stigma and promoting emotional expression within culturally familiar settings (18). Adapting such frameworks to address the psychological consequences of child marriage could strengthen both maternal health services and broader prevention efforts.

This research adds to an increasing body of feminist and trauma-informed research that repositions child marriage as a medical emergency, developmental injustice, and human rights infringement. This work expands phenomenological scholarship by grounding emotional complexity, survivor perspective, and intergenerational insight. It is an affirmation of the importance of qualitative inquiry as a way to uncover the depth of harm and understanding that goes beyond the numbers into the lived experience.

## 7 Limitations and future research

This study used a small, carefully chosen sample from a specific urban community in South India. This limits how the findings can be applied to other social and cultural settings. It does not fully address differences in caste, religion, rural and urban areas, or local law enforcement, which may restrict the analysis's context. Using gatekeepers and referral-based recruitment may have led to selection bias. Participants who were easier to find or willing to share their experiences may differ from those who are hidden or hard to reach.

As a phenomenological inquiry, the study focused on depth and personal meaning. However, this approach limits how broadly the findings can be applied. The researcher's viewpoint also shapes the findings, which raises the risk of bias, even with efforts to remain aware and maintain analytical standards. In addition, relying on people's memories may introduce recall bias. Participants’ accounts could be affected by forgetfulness, current mental states, or new interpretations of past events.

The sensitive nature of the topic may have led to incomplete sharing or social pressure to please, especially regarding sexual coercion, marital issues, and mental health struggles. Cultural stigma around these matters may have made participants hesitant to share their experiences fully. Moreover, the study does not use multiple data sources, such as family members, healthcare providers, or organization records, which could have strengthened the findings.

Future research should expand on these insights with long-term studies that track the psychological, relationship, and identity outcomes of child marriage and adolescent motherhood. Such studies would allow for a deeper understanding of how early experiences affect life paths, including mental health, parenting styles, and economic mobility. Comparative studies across different regions of India and South Asia are also essential to examine how variations in cultural norms, policy applications, and access to services shape experiences.

In addition, further research could mix methods to combine the depth of qualitative data with quantitative analysis, improving both understanding and wider applicability. Studies focusing on the developmental, emotional, and educational outcomes of children born to adolescent mothers would shed light on intergenerational effects. Finally, research evaluating trauma-informed care models, community support programs, and how effective policies are would be crucial for applying research findings to practice and enhancing outcomes for affected groups.

## 8 Conclusion

This study underscores the profound psychological, emotional, and developmental harms of child marriage, reframing it as a systemic violation of children's rights rather than a cultural inevitability. Survivors’ narratives reveal how early marriage operates through coercion, silencing, and identity disruption, often resulting in severe mental health challenges and intergenerational consequences. These harms are further compounded by physical health risks, as evidence shows that child marriage is associated with increased delivery complications and adverse maternal outcomes (40). Sustained by poverty, gender inequality, and restrictive social norms, these experiences are embedded within broader structural conditions that constrain girls’ autonomy and access to care.

At the same time, participants’ accounts reflect resilience and a clear demand for change, emphasizing the importance of education, legal reform, and community accountability. Addressing these harms requires coordinated, trauma-informed responses across healthcare, education, and legal systems, particularly those that are sensitive to the needs of pregnant and postpartum adolescents. Policy-oriented research highlights the necessity of strengthening legal protections, improving access to reproductive and mental health services, and addressing systemic inequities that shape adolescent maternal experiences (41).

Addressing the harms identified in this study requires coordinated structural responses across education, healthcare, and legal systems. Policymakers must prioritize gender-sensitive and trauma-informed approaches within maternal health and adolescent care services, while educators and community leaders play an essential role in creating safe environments that support girls’ autonomy, emotional expression, and continued education. Strengthening legal literacy, expanding mental health support, and fostering community-based advocacy are critical steps toward preventing early marriage and supporting survivors.

Ultimately, this research underscores that child marriage is not only a legal or social issue but a profound psychological and developmental crisis with intergenerational consequences. Listening to the voices of survivors is therefore essential, not only for documenting harm but for guiding meaningful transformation. Their narratives serve not merely as testimonies of suffering but as frameworks for change, reminding policymakers, researchers, and communities that protecting girls’ autonomy, dignity, and emotional wellbeing must remain central to any effort aimed at ending child marriage.

## 9 Recommendations

### 9.1 Trauma-informed mental health support

Develop accessible, youth-friendly mental health interventions, particularly for adolescent mothers suffering from postpartum distress, identity confusion, and emotional isolation.

### 9.2 Survivor-centered education initiatives

Prioritize education for girls through scholarships, retention policies, and community sensitization, and aim to prevent early school dropout and build emotional resilience.

### 9.3 Legal literacy and enforcement

Educate people about child marriage laws, and regular enforcement should be ensured. Protection against the possibility of backlash and the safety of survivors should be the two main concerns, along with the enforcement of the law.

### 9.4 Community-based advocacy

The support of local leaders, teachers, and healthcare workers is essential in shifting norms, elevating survivor voices, and empowering girls’ autonomy through culturally rooted programs.

### 9.5 SDG integration

Align interventions with SDG 5.3 (elimination of harmful practices), SDG 3 (health and wellbeing), SDG 4 (education), SDG 10 (reduced inequalities), and SDG 16 (justice and accountability), so that child marriage is treated as a cross-sectoral crisis.

This study confirms that child marriage is more than a legal matter; it is an emergency in psychology, a travesty in development, and a failure of morality. Transformation needs to start with a hearing from those who have experienced it. Their voices are not only testaments of damage but maps to recovery. To do them justice is to live out their insight, and to create systems of care, justice, and possibility in which no girl is made to make a choice between survival and silence.

## Data Availability

The raw data supporting the conclusions of this article will be made available by the authors, without undue reservation.
